# Specific pattern of maturation and differentiation in the formation of cortical tubers in tuberous sclerosis omplex (TSC): evidence from layer-specific marker expression

**DOI:** 10.1186/s11689-016-9142-0

**Published:** 2016-04-01

**Authors:** Angelika Mühlebner, Anand M. Iyer, Jackelien van Scheppingen, Jasper J. Anink, Floor E. Jansen, Tim J. Veersema, Kees P. Braun, Wim G. M. Spliet, Wim van Hecke, Figen Söylemezoğlu, Martha Feucht, Pavel Krsek, Josef Zamecnik, Christian G. Bien, Tilman Polster, Roland Coras, Ingmar Blümcke, Eleonora Aronica

**Affiliations:** Department of (Neuro) Pathology, Academic Medical Center, Amsterdam, The Netherlands; Department of Pediatrics, Medical University Vienna, Vienna, Austria; Department of Pediatric Neurology, Brain Center Rudolf Magnus, University Medical Center Utrecht, Utrecht, The Netherlands; Department of Pathology, University Medical Center Utrecht, Utrecht, The Netherlands; Department of Pathology, Faculty of Medicine, Hacettepe University, Ankara, Turkey; Department of Neurology, Charles University, 2nd Faculty of Medicine, Motol University Hospital, Prague, Czech Republic; Department of Pathology and Molecular Medicine, Charles University, 2nd Faculty of Medicine, Motol University Hospital, Prague, Czech Republic; Epilepsy Centre Bethel, Krankenhaus Mara, Bielefeld, Germany; Department of Neuropathology, University Hospital Erlangen, Erlangen, Germany; Swammerdam Institute for Life Sciences, Center for Neuroscience, University of Amsterdam, Amsterdam, The Netherlands; Stichting Epilepsie Instellingen Nederland (SEIN), Heemstede, The Netherlands

**Keywords:** Tuberous sclerosis complex, Cortical layer markers, Epilepsy, Neurosurgery, Neuropathology

## Abstract

**Background:**

Tuberous sclerosis complex (TSC) is a multisystem disorder that results from mutations in the TSC1 or TSC2 genes, leading to constitutive activation of the mammalian target of rapamycin (mTOR) signaling pathway. Cortical tubers represent typical lesions of the central nervous system (CNS) in TSC. The pattern of cortical layering disruption observed in brain tissue of TSC patients is not yet fully understood, and little is known about the origin and phenotype of individual abnormal cell types recognized in tubers.

**Methods:**

In the present study, we aimed to characterize dysmorphic neurons (DNs) and giant cells (GCs) of cortical tubers using neocortical layer-specific markers (NeuN, SMI32, Tbr1, Satb2, *Cux2*, *ER81*, and *RORβ*) and to compare the features with the histo-morphologically similar focal cortical dysplasia (FCD) type IIb. We studied a cohort of nine surgically resected cortical tubers, five FCD type IIb, and four control samples using immunohistochemistry and in situ hybridization.

**Results:**

Cortical tuber displayed a prominent cell loss in all cortical layers. Moreover, we observed altered proportions of layer-specific markers within the dysplastic region. DNs, in both tubers and FCD type IIb, were found positive for different cortical layer markers, regardless of their laminar location, and their immunophenotype resembles that of cortical projection neurons.

**Conclusions:**

These findings demonstrate that, similar to FCD type IIb, cortical layering is markedly disturbed in cortical tubers of TSC patients. Distribution of these disturbances is comparable in all tubers and suggests a dysmaturation affecting early and late migratory patterns, with a more severe impairment of the late stage of maturation.

**Electronic supplementary material:**

The online version of this article (doi:10.1186/s11689-016-9142-0) contains supplementary material, which is available to authorized users.

## Background

Tuberous sclerosis complex (TSC) is a genetic disease caused by mutations in the *TSC1* and *TSC2* genes. The two proteins encoded by these genes—tuberin and hamartin—are key regulators of the mammalian target of rapamycin complex 1 (mTORC1) [1]. Therefore, the disease affects multiple organ systems already during development. Brain lesions can be found in about 90 % of patients with TSC and are often associated with intractable epilepsy [2–4]. Only about 40–60 % of all patients with TSC and epilepsy become seizure-free on medication. In a selected subset of TSC patients, epilepsy surgery is considered as a therapeutic option after careful presurgical evaluation [5].

Histology of resected cortical tubers reveals a severely distorted cortex with apparent loss of cortical layers and presence of abnormal cell types (reviewed in [3, 6]). The most common aberrant cell types are dysmorphic neurons (DNs) and bright eosinophilic giant cells (GCs); these are very large cells lying scattered within the cortex and condensed in the white matter. The increase in cellular mass can be explained by the over-activation of anabolic metabolism [7]. Accordingly, the underlying genetic defect results in a constitutive activation of mTORC1 with consequent increase in protein synthesis caused by the production of additional ribosomes and increased rates of messenger RNA translation [8]. The increased knowledge concerning the molecular basis of GC formation has led to novel therapeutic strategies in patients with TSC [9].

However, the origin and phenotype of abnormal cells observed in cortical tubers remain still unclear. It was speculated that in focal cortical dysplasia type IIb (FCD type IIb; [10])—a pathology similar to cortical tubers—these cells arise from radial glial progenitor cells of the ventricular zone representing different stages of maturation [11].

Recently, the recognition of layer-specific neuronal cell markers of the neocortex allowed detailed studies about cortical layer formation. These studies provided insight into the origin and phenotype of cortical neurons in normal cortex but may also contribute to define the pattern of cortical layer disruption in different malformations of cortical development (MCD) in the human brain. These markers specifically labeled subsets of cortical neurons with limited laminar distribution [12]. Furthermore, there is evidence that the identity of each neuron within the neocortex (type of neuron or laminar fate) is predetermined even before the onset of migration [13]. Several studies have been published studying cortical layer markers in FCD. Previous results suggested that FCD samples contain more immature cells than age-matched controls, indicating an activation of progenitor cells that might contribute to the pathophysiology of these lesions [14]. In FCD type IIb, DNs and balloon cells (BC; similar to GCs in cortical tuber) showed distinct expression of layer markers supporting an origin from radial glia and intermediate progenitor cells [14]. Differences in neuronal maturation between FCD subtypes, as well as the existent laminar structure in normal-appearing neurons, led to the conclusion that only a subpopulation of precursor cells is affected [15]. However, another study suggested that these differences might reflect a differential expression of cortical layer markers in various brain regions [16].

In the present study, we aimed to investigate the expression of a panel of layer-specific markers covering all cortical layers (Satb2, *Cux2*, *RORβ*, *ER81*, Tbr1, SMI32, and NeuN) in cortical tubers of patients with TSC compared to perilesional cortex of the same patients, as well as to FCD type IIb or aged- and location-matched controls.

## Methods

### Subjects

In total, we collected nine cortical tubers and five FCD type IIb samples from various epilepsy surgery centers (Department of Pediatric Neurology, Brain Center Rudolf Magnus, University Medical Center Utrecht; Department of Pediatric Neurology, Charles University, 2nd Faculty of Medicine, Motol University Hospital, Prague; Evangelisches Krankenhaus Mara Bethel-Bielefeld, Bielefeld, Germany; Department of Neuropathology, University Hospital Erlangen, Erlangen, Germany) for this study. Extensive presurgical evaluation including 24 h to 5 days video-EEG monitoring, high-resolution MRI, and neuropsychological testing was performed in each patient in order to identify the epileptogenic zone (EZ) before tailored surgical resection. Perilesional cortex (if available in both pathologies) was also included as defined by the lack of dysmorphic neurons and giant/balloon cells in the cortex.

We included an age-matched autopsy control group (*n* = 4). None of these patients had a history of seizures or other neurological diseases.

Tissue was obtained and used in accordance with the Declaration of Helsinki and the AMC Research Code provided by the Medical Ethics Committee and approved by the science committee of the UMC Utrecht Biobank. The local ethical committees of all participating centers gave permission to undertake the study.

### Tissue preparation and immunohistochemistry

The tissue was carefully oriented, cut perpendicular to the pial surface, fixed overnight in 4 % formaldehyde, and routinely processed into liquid paraffin. Sections were cut at 4–6 μm with a microtome (Microm, Heidelberg, Germany) and mounted on positively charged slides (Superfrost + Menzel, Germany). Each specimen was histopathologically examined using hematoxylin and eosin (H & E). In autopsy controls, an additional Nissl staining was performed to calculate overall neuronal densities.

An immunohistochemical examination of all surgical specimens was performed using the following panel of antibodies: NeuN (neuronal nuclei, 1:100, clone A60, Chemicon, Temecula, CA, USA), non-phosphorylated neurofilament H (1:1000, clone SMI32, Sternberger, Lutherville), Satb2 (1:400, clone SATBA4B10, Abcam, Cambridge, UK), and Tbr1 (1:50, polyclonal rabbit, Abcam, Cambridge, UK).

The slides were air-dried overnight at 37 °C. All immunohistochemical stainings were performed with a Ventana semi-automated staining machine (Benchmark ULTRA; Ventana, Illkirch, France) and the Ventana DAB staining system according to the manufacturer’s protocol.

### In situ hybridization

In situ hybridization (ISH) for *Cux2*, *RORβ*, and *ER81* were performed using a 5′–3′ double digoxygenin (DIG)-labeled superior probes (containing locked nucleic acid [LNA] and 2′O-Methyl [2’OMe] RNA moieties; “l” indicates LNA and “m” indicates 2ÓMe RNA; Ribotask ApS, Odense, Denmark):

- *Cux2*: 5’DIG-lAmAmUlTmUmClTmCmUlGmCmAlGmCmAlAmGmGmUmUlT-DIG
- *RORβ*: 5’DIG-lAmAmUlTmUmClCmUmUlGmGmUlTmCmUlAmUmAmAmGlC-DIG
- *ER81*: 5’DIG-lAmAmUlTmUmClTmCmAlTmAmGlTmAmAlTmAmGmCmGlG-DIG

The hybridizations were done on 5-μm sections of paraffin-embedded materials as described previously [17, 18]. The probes were hybridized at 62 °C for 1 h, and the hybridization was detected with alkaline phosphatase (AP)-labeled anti-DIG (Roche Applied Science, Basel, Switzerland). NBT (nitro-blue tetrazolium chloride)/BCIP (5-bromo-4-chloro-3’-indolyphosphate p-toluidine salt) was used as chromogenic substrate for AP. Negative control assays were performed without probes and without primary antibody (sections were blank).

### Quantitative analysis of the immunopositive cells and the ISH signals

To study the alteration of cortical layering, the cortex was divided into three regions: supragranular layers (L1-3, SG), layer 4 plus infragranular layers (L4-6, IG), and deep white matter (>500 μm WM). The number of immunopositive cells was counted at ×10 magnification in five representative fields of L1-3 and L4-6 as well as 10 visual fields of WM. If applicable, the tuber samples were divided into perilesional cortex (PLx) and full-blown tuber (Tb). Perilesional was considered if the cortex showed a normal cortical architecture and no signs of DNs or GCs. A variable degree of giant cells in the white matter was always present.

### Statistical analysis

Statistical analysis was performed on SPSS 21 (IBM, PASW Statistics, USA). The total number of positive cells as well as the ratio of positive cells divided by the total amount of neurons was used for computing the analysis. Descriptive statistics (median, range) and non-parametric testing were performed to evaluate between-group differences. *p* < 0.05 was considered significant.

## Results

### Demographic patient data

Our cohort of epilepsy surgery patients consisted of six female and eight male patients. The control group consisted of two females and two males. Cortical tubers were resected temporally in five cases and frontally in four cases. The somatic mutation in the *TSC* gene was known in three of the patients (*TSC1* = 1; *TSC2* = *2*). The site of FCD type IIb was frontal in four cases and occipital in one. The control group comprised two frontal, one temporal, and one occipital sample. The median age at surgery was 5.00 years (range = 3.00–36.00) in patients with TSC, 12.00 years (range = 1.00–36.00) in patients with FCD type IIb, and 8.50 years (range = 07.00–12.00) in controls.

The first step of the analysis included a general assessment of cortical layering in our different cohorts.

### Cortical layer disruption in TSC cortical tubers and FCD type IIb

**Fig. 1:**
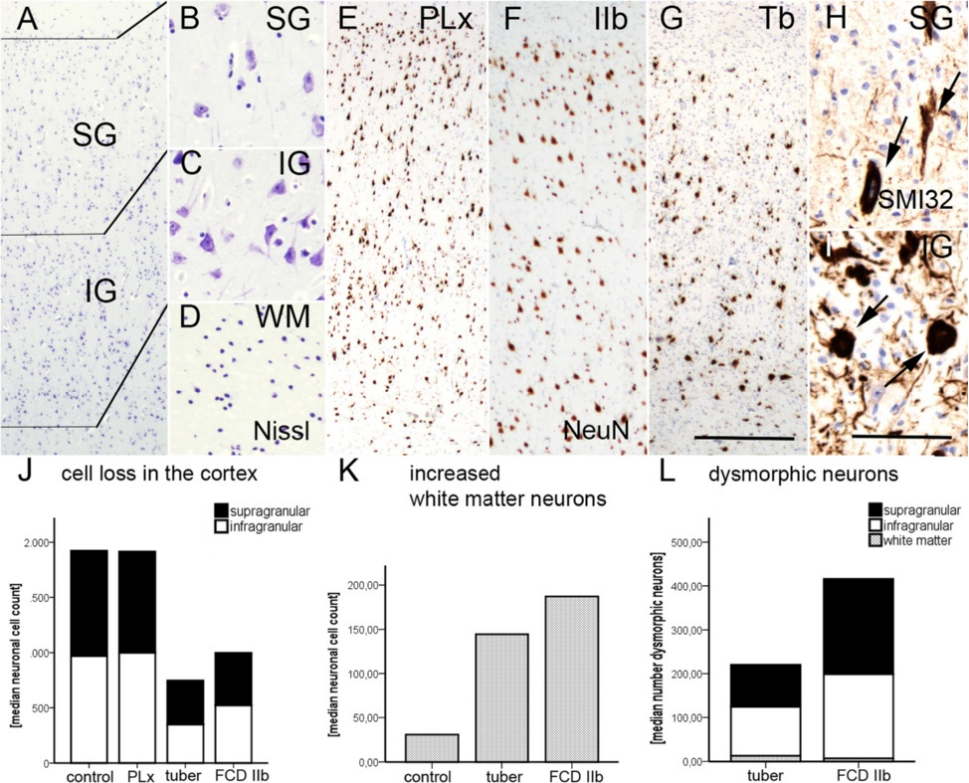
Cortical disorganization in TSC cortical tubers. **a** Normal control cortex with classic 6-layered lamination. **b**, **c** Normally shaped neuronal cells of supra- and infragranular layers. **d** Deep white matter in a control patient. **e** Normal appearing cortex in a perilesional sample. **f** Cortical dyslamination of FCD type IIb in a patient with difficult-to-treat frontal lobe epilepsy. **g** Dysorganization of the cortex in a cortical tuber of a patient with genetically proven TSC. **h**, **i** Dysmorphic neurons (*arrows*) are present throughout the whole cortex. *Scale bar* in **g** equals 500 μm and applies also to **a**, **e**, and **f**. *Scale bar* in **i** equals 200 μm and applies also to **b**–**d**. **j** Loss of neurons throughout all cortical layers in TSC cortical tubers and FCD type IIb. **k** Significant increase in heterotopic neurons in the deep white matter in patients who underwent surgery for FCD Type IIb or cortical tuber resection. **l** A larger amount of dysmorphic neurons can be detected in FCD type IIb compared to TSC cortical tubers. *SG* supragranular layers, *IG* infragranular layers, *WM* white matter, *Co* control, *PLx* perilesional cortex, *Tb* cortical tuber, *IIb* FCD type IIb

In order to determine the extent of dyslamination in TSC cortical tubers, we first addressed the total number of neurons in cortical layers as well as in the white matter from cortical tubers and FCD type IIb (Fig. 1a–i). In our cohort, we detected a significant neuronal cell loss in cortical tubers and FCD type IIb when compared to perilesional tissue and non-epileptic autopsy specimens (Kruskal-Wallis, *p* = 0.000, Table 1, Fig. 1j). In addition, there was a significant increase in heterotopically placed neurons in deep white matter in both pathologies compared to our control samples (Kruskal-Wallis, *p* = 0.033, Table 1, Fig. 1k, Additional file 1: Figure S1). As previously reported [19], DNs are characterized by a pathologic accumulation of non-phosphorylated neurofilament (SMI32) in both FCD type IIb and cortical tubers, and we observed significantly more DNs present in the neocortex of FCD type IIb than cortical tubers (Kruskal-Wallis, *p* = 0.017, Table 1, Fig. 1l, Additional file 1: Figure S1).

**Table 1:** Total cell counts

Pathology	Location	Total neurons [median]	Dysmorphic neurons [median]	Total Tbr1+ cells [median (range)]	Total Satb2+ cells [median (range)]	Total Cux2+ cells [median (range)]	Total RORβ+ cells [median (range)	Total ER81+ cells [median (range)]	Tbr1+ cells per total neurons [%]	Satb2+ cells per total neurons [%]	Cux2+ cells per total neurons [%]	RORβ+ cells per total neurons [%]	ER81+ cells per total neurons [%]
Tuber	SG	399.50 (154.00–899.0)	96.00 (11.00–238.00)	41.00 (0.00–126.00)	63.00 (49.00–161.00)	43.00 (0.00–145.00)	88.00 (5.00–198)	161.00 (99.00–706.00)	8.00	19.00	13.00	23.00	48.00
	IG	348.00 (180.00–739)	111.50 (4.00–204.00)	94.00 (0.00–178.00)	2.00 (0.00–136.00)	13.00 (0.00–112.00)	134.00 (3.00–165.00)	206 (96.00–565.00)	30.00	1.00	4.00	27.00	49.00
FCD IIb	SG	476.00 (439.00–799.00)	217.00 (175.00–275.00)	44.00 (13.00–103.00)	181.00	88.00 (0.00–173.00)	156.00 (15.00–200.00)	373.00 (175.00–799.00)	9.00	23.00	20.00	25.00	85.00
	IG	523.00 (374.00–643.00)	192.00 (113.00–206.00)	61.00 (58.00–287.00)	204.00	76.00 (44.00–123.00)	166.00 (98.00–187.00)	231.00 (182.00–314.00)	12.00	32.00	12.00	26.00	49.00
PLx	SG	916.00 (682.00–2263.00)	0.00	176.00 (0.00–460.00)	193.00 (136.00–315.00)	345.00 (78.00–660.00)	121.00 (0.00–264.00)	227.00 (12.00–652.00)	16.00	19.00	37.00	10.00	27.00
	IG	1000.00 (535.00–1242.00)	0.00	420.00 (246.00–662.00)	14.00 (9.00–215.00)	99.00 (12.00–276.00)	452.00 (120.00–688.00)	519.5 (169.00–987.00)	44.00	3.00	16.00	55.00	64.00
Control	SG	952.00 (299.00–1175.00)	0.00	14.50 (5.00–24.00)	548.00 (420.00–676.00)	367.00	49.00 (35.00–63.00)	65.50 (58.0–73.00)	0.00	36.00	39.00	12.00	19.00
	IG	971.00 (842.00–1495.00)	0.00	321.50 (300.00–343.00)	407.50 (362.00–453.00)	55.00	456.5 (412.00–501.00)	450.00 (407.00–493.00)	23.00	30.00	6.00	50.00	59.00

Overview over the quantitative analysis. Our findings indicate a tendency towards a loss of Satb2+ neurons in layer 5 and an increase of Tbr1+ cells throughout all cortical layers

*SG* supragranular, *IG* infragranular, *FCD* focal cortical dysplasia, *PLx* perilesional cortex

Next, we looked at neuronal fate determination in the remaining neuronal populations utilizing layer-specific markers.

### Satb2 and *Cux2* expression

**Fig. 2:**
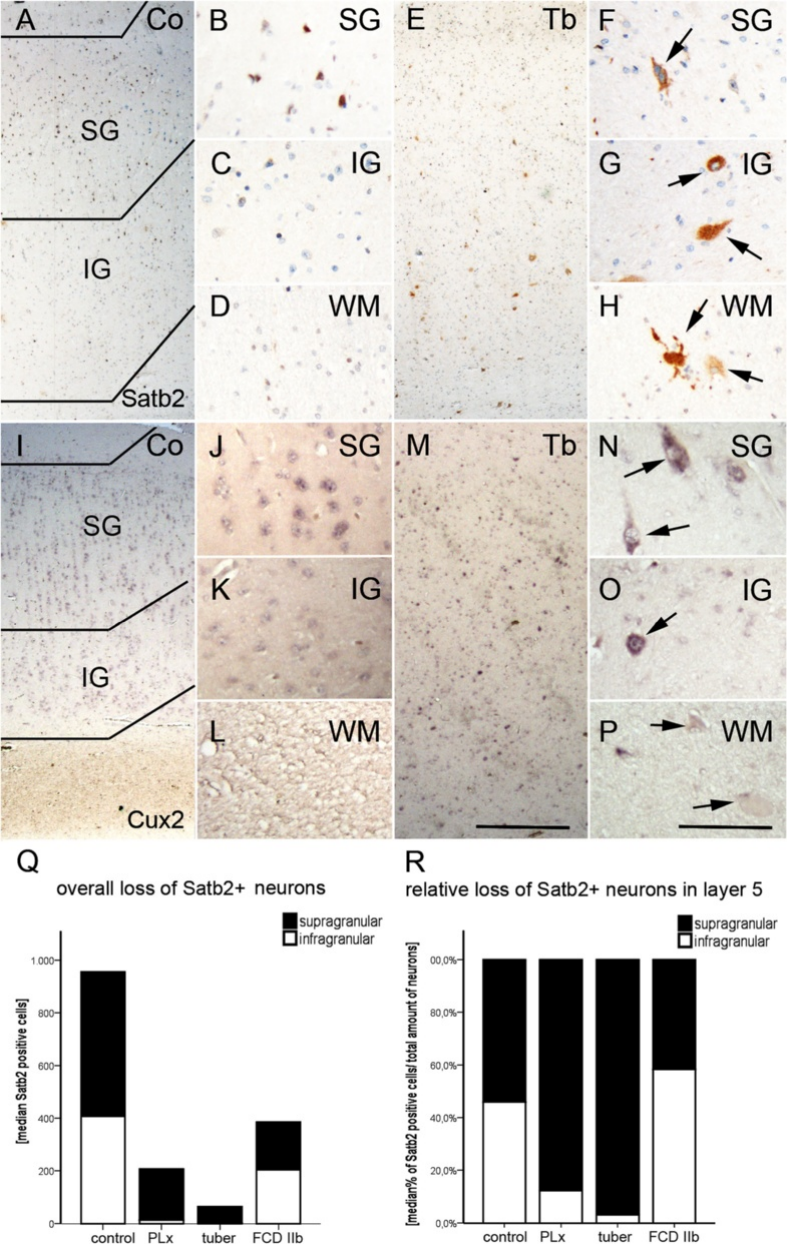
Composition of the upper cortical layers. **a**–**d** Satb2-positive neurons are predominantly in the upper layers of the neocortex. **e** Loss of Satb2+ cells throughout all layers in TSC cortical tubers. **f**–**h** Expression of Satb2 in dysmorphic neurons (*arrows* in **f** and **g**) and giant cells (*arrows* in **h**) of cortical tubers. **i**–**l** Normal distribution of *Cux2* expressing cells in the control cortex. **m** *Cux2*+ cells throughout all layers in TSC cortical tubers. **n**–**p** Expression of *Cux2* in dysmorphic neurons (*arrows* in **n** and **o**) and giant cells (*arrows* in **p**) of cortical tubers. *Scale bar* in **m** equals 500 μm and applies also to **a**, **e**, and **I**. *Scale bar* in **p** equals 200 μm and applies also to **b**–**d**, **f**–**h**, **j**–**l**, and **n**–**l**. **q** Quantitative analysis of Satb2+ neurons revealed significant overall cell loss in all epilepsy surgery specimens. **r** Relative loss of Satb2+ neurons in TSC tissue. *SG* supragranular layers, *IG* infragranular layers, *WM* white matter, *Co* control, *PLx* perilesional cortex, *Tb* cortical tuber, *IIb* FCD type IIb

Neurons in the upper cortical layers were defined by their immunoreactivity for Satb2+ (known to be expressed in L2, L3, L4, and L5; Fig. 2a–h) and *Cux2* (normally expressed in L2 und L3, Fig. 2i–p) [12, 14–16, 20, 21]. Overall, the amount of Satb2+ neurons was decreased in perilesional cortex, cortical tubers, and FCD type IIb compared to non-epileptic autopsy controls (Kruskal-Wallis, *p* = 0.003, Table 1, Fig. 2q, Additional file 1: Figure S1). The proportions of neurons that were Satb2+ and subsequently corrected for the total amount of neurons showed a trend towards loss of Satb2+ neurons in lower cortical layers in TSC tissue (perilesional and tuberal; L5) compared to controls and FCD type IIb (Table 1, Fig. 2r, Additional file 1: Figure S1). However, statistical testing failed to reach significance (Kruskal-Wallis, *p* = 0.083). DNs throughout all cortical layers were Satb2+ in eight cortical tubers and two FCD type IIb samples (Table 2). Except for one sample of FCD type IIb, all giant/balloon cells (GCs/BCs) of layer 1 and in the white matter were strongly positive for Satb2 (Table 2, Fig. 2f–h).

**Table 2:** Expression pattern of cortical layer markers in aberrant cell forms

	TSC cortical tubers		FCD type IIb	
	DN	GC	DN	BC
Satb2	20 %	80 %	15 %	80 %
*Cux2*	80 %	0 %	80 %	0 %
*RORβ*	80 %	10 %	80 %	5 %
Tbr1	0 %	0 %	0 %	0 %
*ER81*	80 %	15 %	80 %	0 %

Only minor differences between TSC cortical tubers and FCD type IIb cells

*TSC* tuberous sclerosis complex, *FCD* focal cortical dysplasia, *DN* dysmorphic neurons, *GC* giant cells, *BC* balloon cells

*Cux2* expression was according to previously published data considered normal in control and perilesional cortex when showing strong expression in L2 and L3 (Fig. 2i–l) [21–23]. In contrast to normal *Cux2* distribution, only in upper layers L2 and L3 in controls, cortical tubers showed less *Cux2+* cells in the upper layers (Kruskal-Wallis, p = 0.023). Corrected for neuronal number, there was however no difference. DNs were strongly *Cux2+* throughout all layers of cortical tubers. Nonetheless, in four out of nine cases, all normally appearing neurons also expressed *Cux2* (Fig. 2m–p). Expression was never detected in giant cells. In FCD type IIb, we observed a similar pattern, although the expression pattern was limited to the upper cortical layers in one case.

### *RORβ* expression

**Fig. 3:**
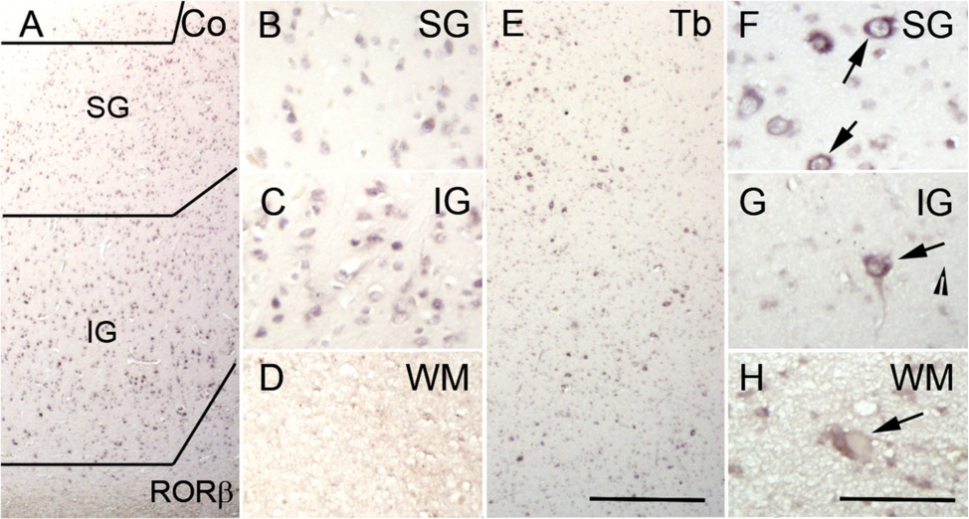
Neurons of L4. **a**–**d** Regular expression pattern of *RORβ* in normal control cortex. **e**–**h** Widespread *RORβ* expression throughout the whole cortex in TSC cortical tubers with positivity in DNs (*arrows* **f**, **g**) in and some GCs (*arrows* in **h**). *Scale bar* in **e** equals 500 μm and applies also to **a** and **e**. *Scale bar* in **h** equals 200 μm and applies also to **b**, **c**, **d**, **f**, and **g**. *SG* supragranular layers, *IG* infragranular layers, *WM* white matter, *Co* control, *Tb* cortical tuber

Neurons of the internal granular layer (L4) can be assessed by ascertaining the expression pattern of *RORβ*. In our control samples and perilesional cortex, expression of *RORβ* was restricted to L4 with some scattered neurons in L6 (Fig. 3a–d). We observed reduced expression of *RORβ* in the lower layers of cortical tubers and FCD type IIb cases (Kruskal-Wallis, *p* = 0.024). However, strong expression was found in DNs of all cortical layers whereas only in two cases of TSC and in one case of FCD type IIb GCs/BCs were slightly stained on ISH (Table 2). Some of the remaining normal-appearing neurons showed also expression in L4-L6 (Fig. 3e–h).

### Tbr1 and *ER81* expression

**Fig. 4:**
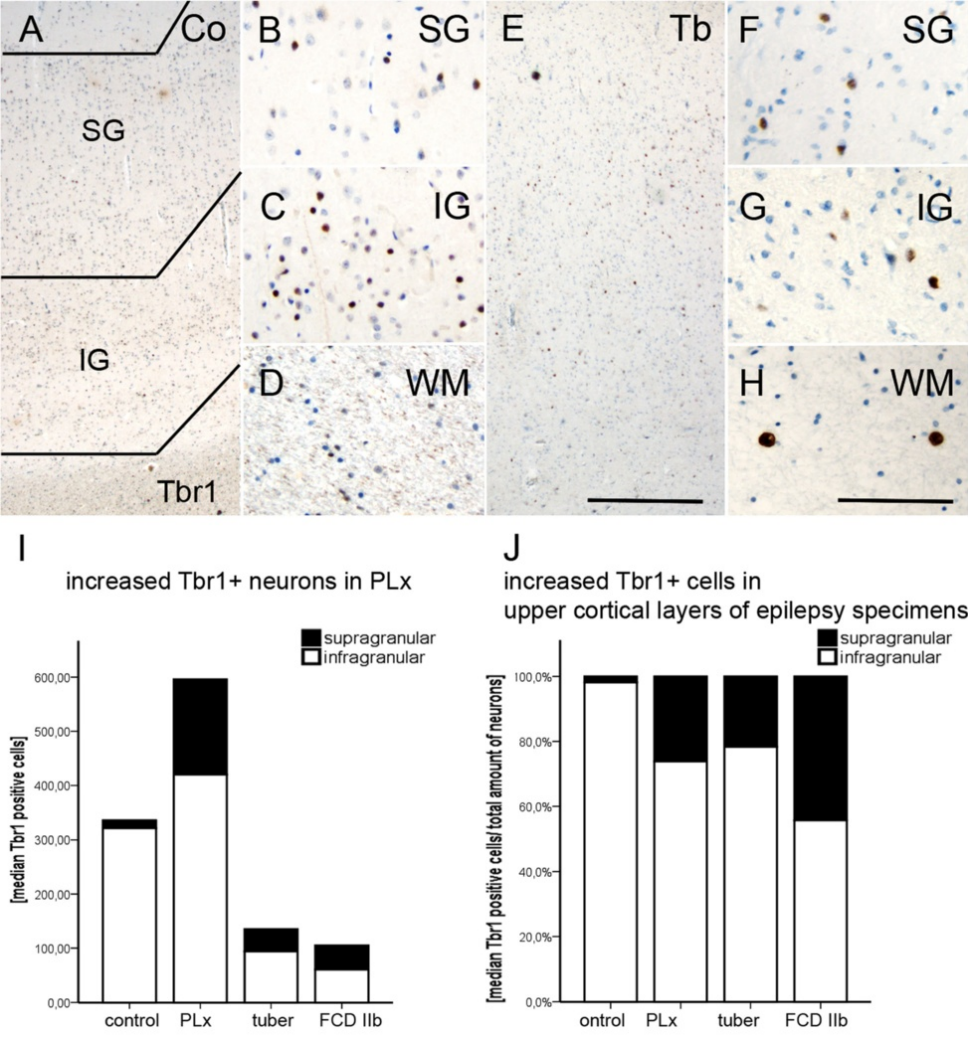
Tbr1+ neurons of the lower cortical layers. **a**–**d** Regular distribution of Tbr1+ neurons in control cortex. **e**–**h** Decrease in overall Tbr1+ neurons in TSC and FCD type IIb. *Scale bar* in **e** equals 500 μm and applies also to **a** and **e**. *Scale bar* in **h** equals 200 μm and applies also to **b**, **c**, **d**, **f**, and **g**. **i** Quantitative increase in Tbr1+ neurons in perilesional cortex (PLx). **j** Quantitative increase of Tbr1+ cells in the upper cortical layers. *SG* supragranular layers, *IG* infragranular layers, *WM* white matter, *Co* control, *PLx* perilesional cortex, *Tb* cortical tuber, *IIb* FCD type IIb

The lower cortical layers (L5/6) can be determined via the expression of Tbr1 and *ER81*. In general, we observed a decrease in the total numbers of Tbr1+ neurons in cortical tubers and FCD type IIb samples (Fig. 4a–d). However, we detected an overall increase in Tbr1+ neurons in the perilesional cortex (TSC and FCD IIb; Kruskal-Wallis, *p* = 0.000; Fig. 4e–i) which failed to reach significance if corrected for the number of neurons. Nonetheless, there was a tendency towards increased number of neurons in the upper cortical layers of cortical tubers and FCD type IIb (Fig. 4j, Addtional file 1: Figure S1); this failed to show significance. DNs and GCs/BCs were negative. The number of cells positive for Tbr1 in the white matter of both pathologies also showed a trend towards increase (Kurskal-Wallis, *p* = 0.089; Table 2).

**Fig. 5:**
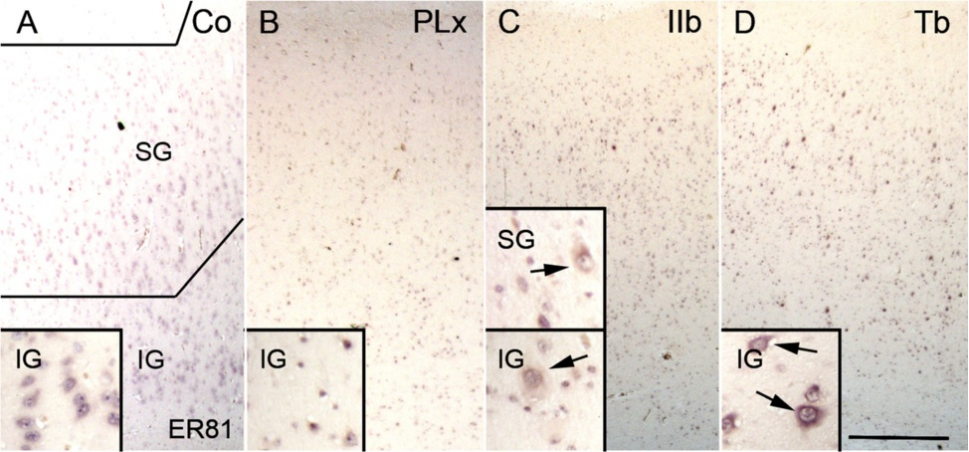
Expression pattern of *ER81*. **a** Strong reactivity for *ER81* in the layers 5/6 in normal controls. **b** No differences in perilesional cortex (PLx). **c** Increased amount of ER81 expressing neurons in the upper layers of the neocortex in FCD type IIb (*arrows*). **d** Strong reactivity in DNs of cortical tubers (*arrows*), however, the constitutive expression pattern in normal appearing-neurons remains intact. *Scale bar* in **d** equals 500 μm and applies also to **a**–**c**. *SG* supragranular layers, *IG* infragranular layers, *Co* control, *PLx* perilesional cortex, *Tb* cortical tuber; *IIb* FCD type IIb

Lastly, we focused on the expression pattern of *ER81*. In autopsy controls and perilesional cortex, the expression of *ER81* was restricted to L5/6 (Fig. 5a/b). This pattern was also found in cortical tubers (Fig. 5d). Nonetheless, in FCD type IIb, we detected a tendency towards the increase of normal-appearing neurons that expressed *ER81* in the upper cortical layers (Kruskal-Wallis, *p* = 0.069, Fig. 5c). DNs in cortical tubers and FCD type IIb showed a strong expression of *ER81*. GCs were slightly stained in three cases. BCs were negative (Table 2). Normal-appearing neurons in the white matter were positive for *ER81*.

For all markers, there was no difference between perilesional cortex of cortical tubers and FCD type IIb.

## Discussion

The development of the human cerebral cortex depends on a precisely orchestrated cascade of events, including proliferation, migration, differentiation, and maturation of neural progenitor cells [24]. The mTOR pathway plays important roles during cortical development, being involved in the regulation of cell growth, proliferation, and survival. Thus, a deregulation of this pathway during corticogenesis can dramatically hamper cortical lamination [25, 26]. Accordingly, aberrant activation of this signaling pathway cascade has been identified in a large spectrum of MCD, TSC being the prototype of this category of MCD which have been referred to as “mTORopathies” [27].

Studying cortical layer formation in malformations of cortical development has recently become possible by the use of neocortical layer-specific markers [12]. The present study provides insights in the pattern of cortical layering in TSC cortical tubers. In summary, we observed an overall loss of neurons in all cortical layers. We observed further a proportional loss of Satb2+ neurons in lower cortical layers. In contrast, *Cux2* was expressed in all cortical layers of tubers and FCD type IIb. In addition, we detected an increase of Tbr1+ and *ER81* expressing cells in upper cortical layers. The distinct patterns of disruption of upper and lower cortical layers indicate that dysmaturation of the cortex is a key feature also in TSC brain. However, our data suggests that the upper cortical layers are more severely hampered due to the remnants of neurons that were supposed to relocate in deeper cortical layers implying a more serious impairment of their late stage of maturation.

### Satb2, Cux2, RORβ Tbr1, and ER81 expression patterns in TSC: loss of projection neurons in cortical tubers

Satb2 is an AT-rich DNA-binding protein that has been shown to represent a postmitotic determinant for upper-layer neuron specification in the neocortex [28, 29]. Previous studies reported that Satb2+ cells are present in the layers 2 and 3. Additionally, it was suggested that they disappear after 3 months of age in the human brain [20, 29]. On the contrary, it was also reported that Satb2+ cells remain as a subpopulation in the upper cortical layers of the human brain but also extending towards layer 5 (having a predisposition for the frontal lobe) [30, 31]. In our study, expression of Satb2 was detected in L2, 3, and 5 in both perilesional samples and postmortem controls. In TSC cases (tuber and peritubetal cortex), we observed a lower number of Satb2+ neurons compared to controls and there was a tendency towards a loss of Satb2+ neurons in L5 compared to both controls and FCD type IIb. These findings are therefore suggestive for a specific loss of cells in a subpopulation of cortical neurons in TSC indicating an abnormal formation of pyramidal cells. However, abnormal Satb2 expression in the superficial region of the neocortex has been previously reported in FCD type II, showing a different pattern compared to hemimegalencephaly, which is characterized by a diffused Satb2 cortical expression pattern [20]. Altered Satb2 expression patterns by random migration were also reported in various lissencephalies [29]. Another study focusing on FCD showed Satb2+ neurons diffusely distributed in the cortex and/or the white matter of FCD type II cases, without differences in Satb2+ cell densities between FCD type IIa and FCD type IIb [15]. In addition, we observed Satb2 expression in both DNs and GCs/BCs. However, previous studies evaluating Satb2 expression in FCD case do not specify the cell subtypes (DNs, BCs, normal-appearing neurons); thus, comparison cannot be performed.

Cux proteins, putative markers of upper cortical layer neurons [22], have been shown to be critically involved in the regulation of normal dendritic development of L2-3 neurons [23]. In agreement with previous studies in human neocortex [21], in both control and TSC-perituberal cortex, *Cux2* mRNA was expressed in L2-3. However, in cortical tubers, this pattern was disrupted and the expression of *Cux2* was extensively heterogeneous with variable expression in normal-looking neurons and DNs (also in FCD IIb), but not in GCs in our cohort. It seems therefore that the fate determination of these neurons is altered and they stay diffusely behind throughout the whole cortex without finding its proper way towards the upper layers. In addition, expression of *Cux2* mRNA in both abnormal and DNs has been also reported in a larger cohort of FCD cases, without revealing any differences between type IIa and IIb [21]. In contrast, Cux1 protein expression has not been detected in DNs [14].

The *RORβ* gene is known as a selective marker for internal granular layer (L 4) of mammalian neocortex [12, 32, 33]. According to previous studies [16, 21, 33], also in our cohort of controls and perilesional TSC cortex, expression of *RORβ* mRNA was intensely expressed in L4. In cortical tubers remaining normal-appearing neurons maintained the expression *RORβ* in L4. These results are partly in line with the reported pattern in FCD II cases [16, 21]. In our cohort, similar pattern was observed in tubers with different cortical localization, whereas differences between frontal and temporal resections have been reported in FCD cohorts [16]. *RORβ* expression was also detected in DNs, distributed throughout all layers within the tuber. Expression of *RORβ* mRNA in DNs has been also reported in FCD type II [21].

Tbr1 and *ER81* are well-known neocortical layer-specific markers used to identify the lower cortical layers (L5/6) [16, 34, 35]. Tbr1 gene encodes a transcription factor necessary for the differentiation of neural stem cells in developing brain and highly expressed in glutamatergic early-born cortical neurons [12, 34, 36, 37]. According to these previous studies, Tbr1 was prominently expressed in the lower cortical layers. In both TSC and FCD cases, we observed a lower number of Tbr1+ neurons compared to controls and perilesional cortex; however, if corrected for the total number of neurons, there was a tendency towards an increased number of neurons in the upper cortical layers and in the white matter of epilepsy surgery specimens. Abnormal distribution of Tbr1+ cells throughout the cortex has been reported in both FCD and hemimegalencephaly [14, 20], and localization of Tbr1+ cells in the superficial cortical region has been observed in FCD type II cases [20]. In our cohort, we also observed an increase in Tbr1+ neurons in the perilesional cortex. Interestingly, increasing evidence indicates the extension of structural and molecular abnormities in the perilesional cortex which is an integral part of the epileptic network in TSC brain [38–41].

The transcription factor *ER81* represents another marker of the projection neurons of L5-6 [12, 42, 43]. *ER81* mRNA expression pattern was preserved in the perilesional cortex. In cortical tubers, the constitutive expression pattern in normal-appearing neurons was relatively preserved; however, strong *ER81* expression was detected in DNs throughout the dysplastic cortex. Expression of Er81 in both DNs and normal-appearing neurons has been also observed in FCD type II cases [21, 35]. In agreement with previous reports [16, 35], ER81 expression was also observed in normal-appearing neurons in the white matter of all surgical epilepsy specimens and in the upper cortical layer of FCD cases [35]. Nonetheless, these findings should be carefully interpreted in the light of an overall reduction of neurons in cortical tubers and FCD type IIb [44].

How can the altered layer-specific marker expression be interpreted in the context of the pathogenesis of the lesions? The data in TSC and FCD type II suggest an aberrant migration with a greater impairment in late stage cortical development as a common etiopathogenic mechanism underlying the pattern of cortical layer disruption observed in both lesions. The relative layer preservation reported in other types of FCD (such as FCD IIIa; [16]) indicates more a lesion-induced cause rather than simply the effect of seizure activity. The constitutive activation of mTOR (observed in both TSC and FCD type II) as a consequence of the underlying genetic defects may represent the common molecular link resulting in abnormal cell morphology, migration, and differentiation during corticogenesis and contributing to the aberrant protein expression observed in these lesions.

### Cortical layer disruption in TSC cortical tubers

Evaluation of the neuronal densities (with a pan-neuronal maker) in our cohort of TSC patients revealed significant neuronal cell loss in cortical tubers compared to both perilesional tissue and non-epileptic autopsy controls. A similar reduction of neuronal densities is also detected in FCD type IIb cases. Our findings are in line with previous stereological studies performed in FCD cases (including both pediatric and adult cases). These already showed lower cortical neuronal densities in the region of dysplasia (particularly in FCD type IIb) compared to the adjacent cortex or non-epilepsy controls, regardless of age and cortical localization [35, 44]. Whether reduced neuronal densities may simply reflect cortical volume, expansion is still a matter of discussion since archival paraffin blocks are not suitable for accurate evaluation of the total volume of the dysplastic region [35, 45]. However, in TSC patients (but not in FCD patients), lower gray matter NeuN densities have been shown to be correlated with reduced MRI gray and white matter volumes, suggesting a reduced neuronal production during corticogenesis (failure of progenitor cell proliferation) [46]. However, we cannot exclude selective vulnerability of neurons in which the mTOR signaling is activated. Accordingly, hyperactivation of this pathway has been shown to contribute to cell damage, promoting aging-related changes (reviewed in [47]. Our recent observations suggest that the abnormal activation of mTOR may contribute to apoptosis signaling pathways and premature activation of mechanisms of neurodegeneration in both FCD type II and TSC [48]. Interestingly, although DNs were detected in all cortical layers in both FCD type IIb and TSC cortical tubers, their number was lower in cortical tubers compared to FCD.

We also observed a significant increase in heterotopic neurons in the deep WM in both FCD and TSC specimens compared to controls. This, however, represents a common finding in epilepsy cases, including FCD [46, 49], as well as mild malformation of cortical development (mMCD) [50]. Moreover, the mechanisms underlying the presence of heterotopic WM neurons (i.e., local migratory failure or to incomplete regression of subplate neurons), their relationship with seizure activity, and their role in epileptogenesis remain still unclear [50, 51].

## Conclusions

**Fig. 6:**
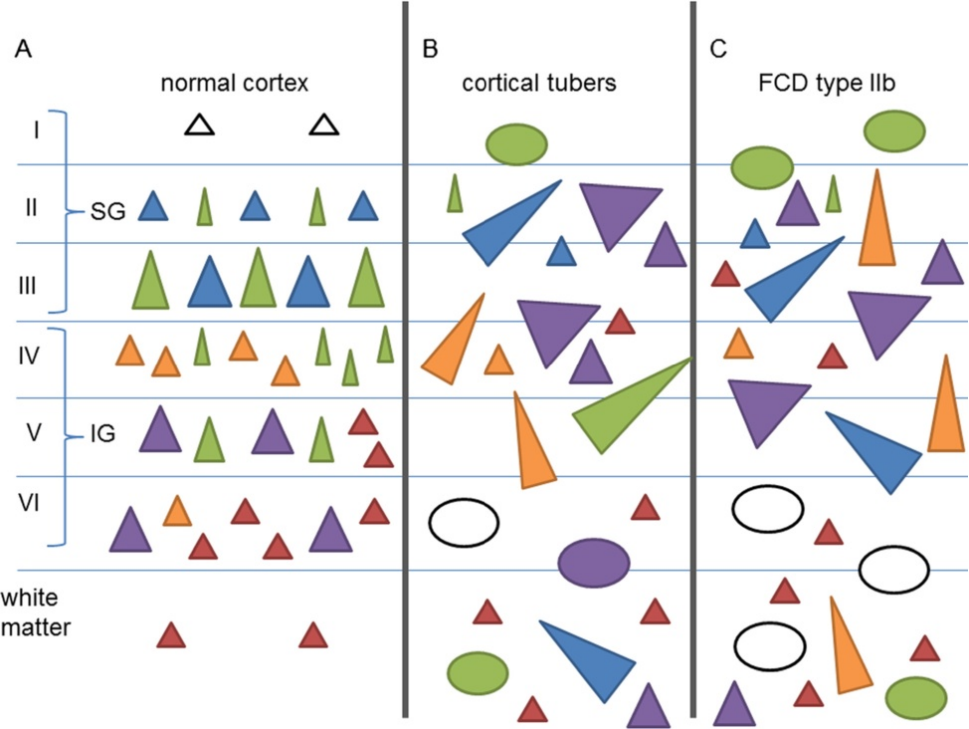
Schematic overview of layer marker expression. **a** Cortical layer markers are expressed in normal cortex as follows: *Cux2*: layers II and III (*blue*); Satb2: layers II, III, IV, and V (*green*); *RORβ* in layer IV and to a lesser extent layer VI (*orange*); *ER81* in layers V and VI (*purple*), and Tbr1 in layers V and VI and few white matter neurons (*red*). **b**, **c** Expression pattern of cortical layer markers in tubers and FCD type IIb: dysmorphic neurons strongly expressed *Cux2*, *RORβ*, and *ER81* and to a lesser extent Satb2 throughout all layers of the cortex. They did not express Tbr1. Giant cells of cortical tubers often expressed Satb2, *ER81*, or none of the markers. Balloon cells of FCD type IIb showed a similar pattern although to a lesser extent, BCs did more frequently express none of the markers. Trends observed in normal-appearing neurons: *Cux2*+ and Satb2+ neurons were less in cortical tubers. The *RORβ+* cells of layer IV were reduced in both pathologies. The expression of Tbr1 and *ER81* was increased in the upper cortical layers and was also present in normal-appearing neurons of the white matter. *Triangles* neurons, *circles* giant/balloon cells, *SG* supragranular layers, *IG* infragranular layers

In summary, our findings provide insight in the abnormal pattern of lamination observed in cortical tubers (summarized in Fig. 6). We provide evidence for an excess of early stage migrational neurons (ER81 and Tbr1) shifting towards the upper layers and therefore hampering late stage migration albeit only the subpopulation of CUX2 expressing cells being affected and leaving the Satb2 population intact. The existence of hidden cortical lamination involving normal-looking neurons has also been recently reported in FCD type II [21]. Moreover, similar to FCD type II [16, 21, 35], DNs (and to a lesser extent GCs) in cortical tubers displayed variable expression of different cortical layer markers (Satb2, Cux2, RORβ, and Er81) regardless of their laminar location, supporting their altered migratory pattern. The immunophenotype of DNs in TSC, similar to FCD type II cases [35], resembles that of cortical projection neurons and suggests an alteration of a selected population of intermediate progenitor cells. Several hypotheses concerning the contribution of DNs and BCs/GCs to local pattern of cortical lamination have been proposed [52, 53], suggesting that both cell-autonomous and non-cell-autonomous mechanisms may be operative during corticogenesis in TSC brain.

## Additional file

Additional file 1: Figure S1.
**A**. Loss of neurons throughout all cortical layers in TSC cortical tubers and FCD type IIb. **B**. A larger amount of dysmorphic neurons can be detected in FCD type IIb compared to TSC cortical tubers. **C**. Significant increase in heterotopic neurons in the deep white matter in patients who underwent surgery for FCD Type IIb or cortical tuber resection. **D**. Quantitative analysis of Satb2+ neurons revealed significant overall cell loss in all epilepsy surgery specimens. **E**. Relative loss of Satb2+ neurons in TSC tissue. **F**. Quantitative increase in Tbr1+ neurons in perilesional cortex (PLx). **G**. Quantitative increase of Tbr1+ cells in the upper cortical layers. (TIF 26642 kb)
