# Supplementary figures and images for: Specific pattern of maturation and differentiation in the formation of cortical tubers in tuberous sclerosis omplex (TSC): evidence from layer-specific marker expression
Source: J Neurodev Disord. 2016 Apr 1;8:9. doi: 10.1186/s11689-016-9142-0 (PMC4818922; doi:10.1186/s11689-016-9142-0)

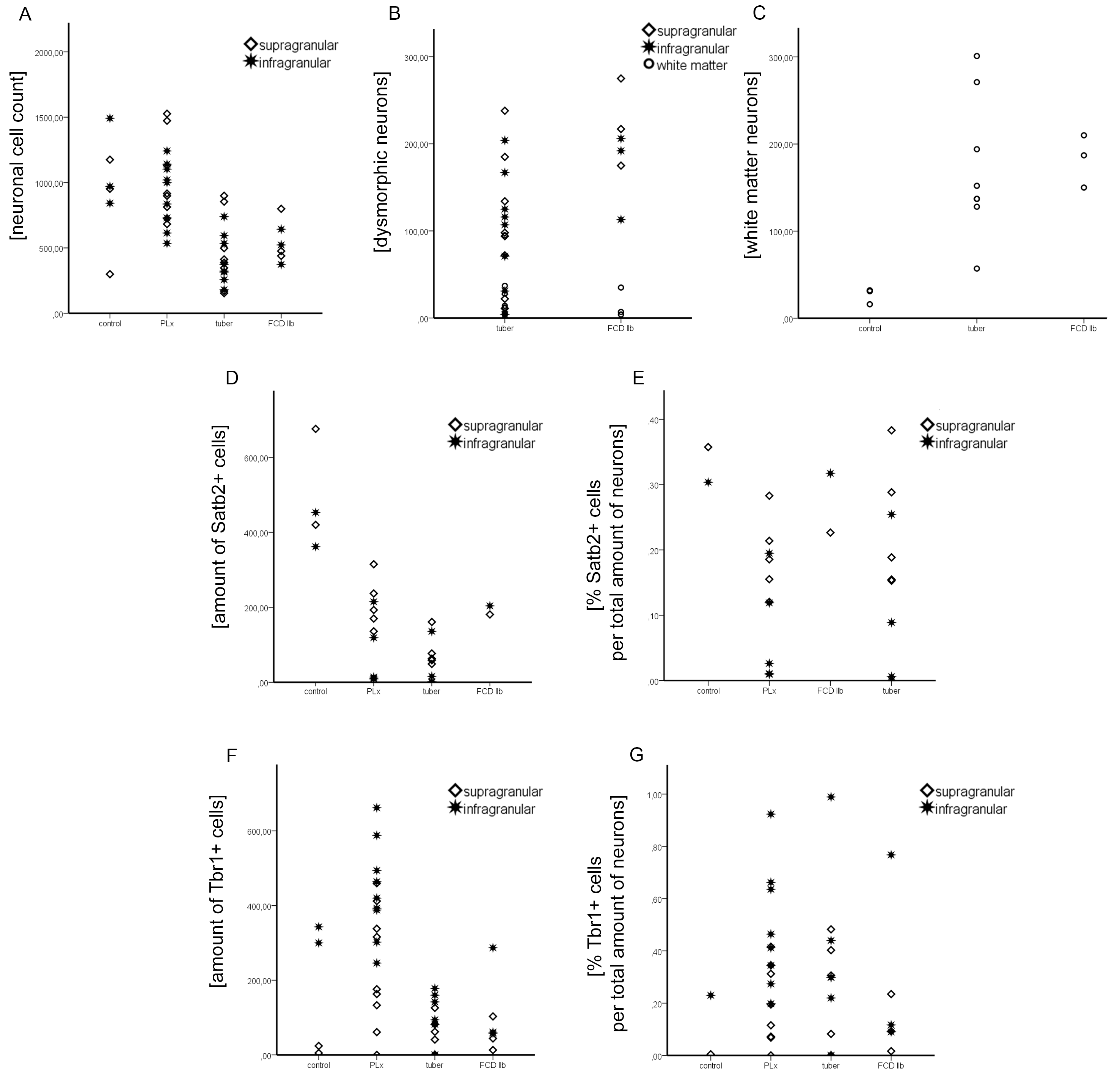

Supplement: Additional file 1: Figure S1. — A. Loss of neurons throughout all cortical layers in TSC cortical tubers and FCD type IIb. B. A larger amount of dysmorphic neurons can be detected in FCD type IIb compared to TSC cortical tubers. C. Significant increase in heterotopic neurons in the deep white matter in patients who underwent surgery for FCD Type IIb or cortical tuber resection. D. Quantitative analysis of Satb2+ neurons revealed significant overall cell loss in all epilepsy surgery specimens. E. Relative loss of Satb2+ neurons in TSC tissue. F. Quantitative increase in Tbr1+ neurons in perilesional cortex (PLx). G. Quantitative increase of Tbr1+ cells in the upper cortical layers. (TIF 26642 kb) [file 11689_2016_9142_MOESM1_ESM.tif]
